# A Comparison of the Effects of Different Positive End-Expiratory Pressure Levels on Respiratory Parameters During Prone Positioning Under General Anaesthesia: A Randomized Controlled Trial

**DOI:** 10.7759/cureus.68693

**Published:** 2024-09-05

**Authors:** Yazhini Shanmugam, Rajagopalan Venkatraman, Aravindhan KY

**Affiliations:** 1 Anaesthesiology, SRM Medical College and Hospital, Chennai, IND

**Keywords:** dynamic compliance, hemorrhage, mean arterial pressure (map), positive end expiratory pressure (peep), prone position

## Abstract

Background and objective

In general anesthesia, for certain surgical procedures in the prone position, patients often face increased airway pressures, reduced pulmonary and thoracic compliance, and restricted chest expansion, all of which can affect venous return and cardiac output, impacting overall hemodynamic stability. Positive end-expiratory pressure (PEEP) is used to address these issues by improving lung recruitment and ventilation while reducing stress on lung units. However, different PEEP levels also present risks such as increased parenchymal strain, higher pulmonary vascular resistance, and impaired venous return.

Proper positioning and frequent monitoring are key to ensuring adequate oxygenation and minimizing complications arising from prolonged periods in the prone position. This study aimed to evaluate the effects of different PEEP levels (0 cmH_2_O, 5 cmH_2_O, and 10 cmH_2_O) in the prone position to determine the optimal setting for balancing improved oxygenation and lung recruitment against potential adverse effects. The goal is to refine individualized PEEP strategies beyond what is typically outlined in standard PEEP tables. We endeavored to examine the impact of different PEEP levels during pressure-controlled ventilation (PCV) on arterial oxygenation, respiratory parameters, and intraoperative blood loss in patients undergoing spine surgery in a prone position under general anesthesia.

Methodology

This randomized, single-blinded, controlled study enrolled 90 patients scheduled for elective spine fixation surgeries. Patients were randomized into three groups: Group A (PEEP 0), Group B (PEEP 5), and Group C (PEEP 10). Standardized anesthesia protocols were administered to all groups, with ventilation set to pressure-controlled mode at desired levels. PEEP levels were adjusted according to group allocation. Arterial blood gases were measured before induction, 30 minutes after prone positioning, and 30 minutes post-extubation. Arterial line insertion was performed, and dynamic compliance, mean arterial pressure (MAP), heart rate (HR), and intraoperative blood loss were recorded at regular intervals. Data were analyzed using SPSS Statistics version 21 (IBM Corp., Armonk, NY).

Results

Arterial oxygenation was significantly higher in Groups B (PEEP 5) and C (PEEP 10) compared to Group A (PEEP 0) at both 30 minutes post-intubation and post-extubation. Specifically, at 30 minutes post-intubation, arterial oxygenation was 142.26 ±24.7 in Group B and 154.9 ±29.88 in Group C, compared to 128.18 ±13.3 in Group A (p=0.002). Similarly, post-extubation arterial oxygenation levels were 105.1 ±8.28 for Group B and 115.46 ±15.2 for Group C, while Group A had levels of 97.07 ±9.90 (p<0.001). MAP decreased significantly in Groups B and C compared to Group A. Dynamic compliance was also improved in Groups B and C. Furthermore, intraoperative blood loss was notably lower in Group C (329.66 ±93.93) and Group B (421.16 ±104.52) compared to Group A (466.66 ±153.76), and these differences were statistically significant.

Conclusions

Higher levels of PEEP (10 and 5 cmH_2_O) during prone positioning in spine surgery improve arterial oxygenation, dynamic compliance, and hemodynamic stability while reducing intraoperative blood loss. These findings emphasize the importance of optimizing ventilatory support to enhance patient outcomes during prone-position surgeries.

## Introduction

Anesthesia is a critical component of surgical procedures and is administered to patients positioned in various ways to optimize surgical access and patient safety [1]. One such position is the prone position, which is often required for specific surgical interventions. This position can lead to several physiological changes and complications, particularly affecting the respiratory system. In the prone position, patients may experience increased airway pressures and decreased pulmonary and thoracic compliance, which can significantly impact chest expansion and abdominal cavity compression [2,3]. This in turn will lead to a significant decrease in dynamic compliance and an increase in peak airway pressure during anesthesia in the prone position.

Functional residual capacity (FRC) is reduced during general anesthesia, mainly due to changes in thoracic blood volume, elevated abdomen pressure, and a decrease in inspiratory muscle tone [4]. The FRC decreases by around 20% as the patient lies on the operating table and moves from an upright to a supine posture [5], with an additional 10% reduction observed at the onset of anesthesia [6]. Furthermore, atelectasis development is linked to general anesthesia. High tidal volumes, high plateau pressures, and the lack of positive end-expiratory pressure (PEEP) are characteristics of general anesthesia ventilation methods, which might cause inflammatory damage, even in patients without any underlying medical conditions [7]. These tactics have the potential to cause atelectasis, which exacerbates the inflammatory damage. Intraoperative gas exchange is compromised, hypoxemia occurs after surgery, and secretion clearance is compromised, increasing the risk of infection [8,9]. Applying PEEP as an atelectasis-reduction strategy may reduce the risk of unfavorable surgical outcomes such as pneumonia, respiratory failure, and death.

Lung protective ventilation techniques, which include low tidal volume, recruitment maneuvers, and PEEP, have been shown to be beneficial in preventing atelectasis [9,10]. These techniques are helpful not only in critical care units but also when a patient is under general anesthesia and in a prone position. Adverse consequences such as hypotension, lower cardiac output, volutrauma, and barotrauma to the injured lungs have been linked to the administration of PEEP [11]. It is not clear what the ideal PEEP is for anesthetized patients in the prone position to avoid atelectasis without causing negative side effects [12]. Hence, this study aimed to compare the effect of three different PEEP levels on respiratory parameters in anesthetized patients in a prone position. The primary objective was to compare arterial oxygenation at pre-induction, 30 minutes after prone positioning, and 30 minutes after extubation when three different amounts of PEEP were applied. The secondary objectives were to compare intraoperative blood loss and hemodynamic parameters in spine surgeries.

This study was previously presented at the 70th Indian Society of Anaesthesiologists' National Conference, on November 24, 2023, at Gurugram.

## Materials and methods

**Figure 1:**
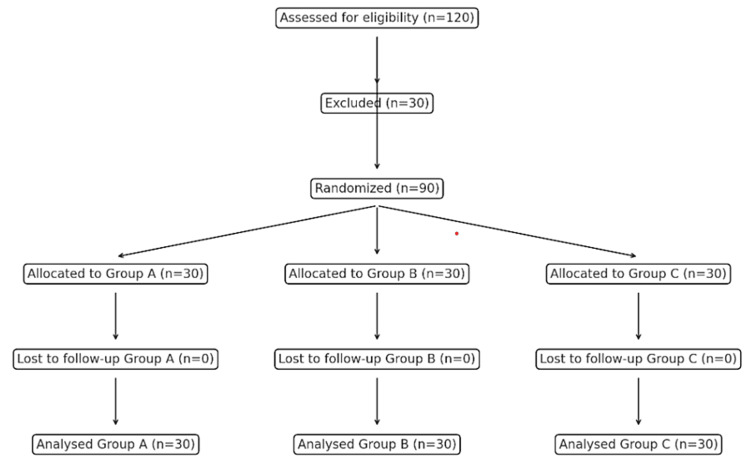
CONSORT diagram depicting the selection of participants CONSORT: The Consolidated Standards of Reporting Trials

This randomized, single-blinded controlled trial was conducted at a medical college hospital between 2022 and 2023 after institutional ethical committee approval and Clinical Trials Registry - India registration (CTRI/2021/11/038109). Ninety patients scheduled for spine surgeries in a prone position were randomly selected and divided into three groups by randomization: Group A: No PEEP, Group B: PEEP of 5, and Group C: PEEP of 10. The inclusion criteria were as follows: patients aged 18-65 years, the American Society of Anesthesiologists (ASA) physical status classification I and II, and scheduled for elective spine instrumentation surgery. Patients with cardiovascular heart diseases, left ventricular ejection fraction <40%, hepatic or renal dysfunction, and patients who refused to participate were excluded. All eligible patients underwent pre-anesthetic evaluation in the clinic, where they were thoroughly assessed and counseled before enrolment in the study. The study adhered to the tenets of the Declaration of Helsinki, 2013, and followed the guidelines outlined in the Consolidated Standards of Reporting Trials (CONSORT) (Figure 1).

The randomization of patients was performed using a computer-generated random number generator. Block randomization was performed with random block sizes of four. To maintain allocation concealment, each patient was assigned a unique number, and this number, along with the assigned group, was recorded on a piece of paper. The paper was then placed inside an opaque envelope and sealed. On the day of the surgery, the envelopes containing the group allocation information were opened by an individual who was not affiliated with the study. He had set the PEEP according to the group involved, ensuring that it remained concealed from the view of others. The anesthesiologist performing the case was blinded to the group involved and the values were recorded.

General anesthesia was standardized in all the groups. The patients were premedicated with alprazolam on the previous night and two hours before surgery. Patient monitoring included pulse oximetry, non-invasive blood pressure (NIBP) measurement, ECG recording, and capnography. All patients were premedicated with glycopyrrolate 0.2 mg and 4 mg ondansetron intravenously (IV). Intraoperative analgesia was provided with 0.15 mg/kg of morphine. The induction agent was 2 mg/kg of propofol, and intubation was facilitated with 0.1 mg/kg of vecuronium. Anesthesia was maintained using a mixture of 50% O_2_ and 50% air with 1-2% sevoflurane. Patients were ventilated with pressure-controlled ventilation (PCV) to achieve a tidal volume of 6 ml/kg. The respiratory rate was maintained at 14 breaths per minute. The pressure and respiratory rate were adjusted to keep end-tidal CO_2_ (ETCO_2_) between 36 and 40. The level of PEEP was adjusted according to the assigned group.

Arterial oxygen saturation (pO_2_) was monitored at three different time points: before induction (T1), 30 minutes after prone positioning (T2), and 30 minutes after extubation. An arterial line was inserted after the patient was shifted to the operating room. Arterial blood gas (ABG) analysis was performed at the three designated time points to assess pH, pO_2_, and pCO_2_. Peak pressure, plateau pressure, mean pressure, dynamic compliance, heart rate (HR), and mean arterial pressure (MAP) were measured every 15 minutes during the surgery. Total intraoperative blood loss was calculated at the end of surgery in all three groups. Hypotension, defined as a reduction in MAP by more than 20% from baseline, was initially treated with crystalloids and ephedrine in increments of 6 mg if necessary [13].

Every participant was seen the day before surgery, and written informed consent was obtained if the patient and/or the patient's legal guardian approved the patient's involvement in the study. The patient was not included in the study if they or their legal guardian refused to give consent. In line with best clinical practices, every member of the research team received training on how to acquire informed consent. SPSS Statistics version 21 (IBM Corp., Armonk, NY) was used for analysis and the gathered data were imported into Microsoft Excel. One-way ANOVA was conducted to compare the effects of PEEP across three groups, and chi-square tests were used to compare categorical data.

## Results

The distribution of demographic profiles was similar in all three groups, as shown in Table 1. The mean age of the participants was 47.56 years with a standard deviation of 12.348 years. Females comprised 54.4% (n=49) of the study participants. The mean BMI of the participants was 24.24 kg/m^2^ with a standard deviation of 3.22 kg/m^2^. The three study groups were comparable and there was no statistically significant difference.

**Table 1:** Demographic parameters and perioperative hemodynamic values (n=90) P<0.05 indicates statistical significance ASA PS: American Society of Anesthesiologists physical status; BMI: body mass index; SD: standard deviation

Variables	Group A (n=30)	Group B (n=30)	Group C (n=30)	P-value
Age, years, mean ±SD	48.4 ±12.93	43.63 ±11.88	50.63 ±11.5	0.079
Gender, F/M, n	14/16	16/14	19/11	0.42
BMI, Kg/m^2^,mean ±SD	24.53 ±2.34	23.76 ±3.33	24.43 ±3.77	0.60
ASA PS, I/II, n	18/12	16/14	19/11	0.72
Duration of surgery, minutes, mean ±SD	128.66 ±24.99	128 ±21.96	122 ±21.55	0.46

Table 2 presents the mean heart rates of participants across three groups, ranging from 62 to 112 beats per minute (BPM) during anesthesia. The intergroup variation in heart rate was found to be statistically non-significant.

**Table 2:** Mean heart rate (BPM) variability of the participants in the three groups (n=90) P<0.05 indicates statistical significance BPM: beats per minute; SD: standard deviation

Time, minutes	Heart rate, BPM, mean ±SD			P-value
	Group A (n=30)	Group B (n=30)	Group C (n=30)	
Baseline	77.86 ±13.12	79.45 ±13.27	75.5 ±8.53	0.57
15	77.73 ±14.09	78.32 ±13.87	76.73 ±10.72	0.15
30	77.43 ±11.11	76.43 ±10.56	77.76 ±12.18	0.07
45	75.5 ±11.18	76.4 ±9.01	76.76 ±8.62	0.13
60	72.86 ±10.29	73.82 ±11.56	74.76 ±8.20	0.20
75	75.6 ±11.03	76.1 ±10.52	76.2 ±8.61	0.25
90	74.66 ±11.21	74.96 ±10.08	74.43 ±10.23	0.67
105	74.63 ±9.86	75.1 ±9.92	73.96 ±10.85	0.91
120	73.7 ±9.61	74.9 ±9.12	74.5 ±12.83	0.60

Table 3 describes the MAP changes in the three group measures during anesthesia. The changes in MAP during anesthesia were statistically significant between the participants (p<0.05), but not the baseline. Similarly, when comparing the groups, the changes in MAP were statistically significant between Group A and Group C compared to Group A with Group B and Group B with Group C. MAP showed a significant reduction in PEEP 10 and PEEP 5 than PEEP 0.

**Table 3:** Comparison of MAP during anesthesia between the groups (n=90) *Statistically significant (p<0.05 indicates statistical significance) MAP: mean arterial pressure; SD: standard deviation

Time, minutes	MAP, mmHg, mean ±SD			P-value
	Group A (n=30)	Group B (n=30)	Group C (n=30)	
Baseline	80.86 ±13.12	81.83 ±8.82	80.53 ±10.71	0.97
15	84.6 ±10.45	78.46 ±5.20	77.5 ±11.21	0.008*
30	80.36 ±8.63	77.9 ±10.48	74.43 ±9.43	0.05*
45	82.83 ±10.05	77.63 ±6.50	74.7 ±7.96	<0.001*
60	80.76 ±8.55	77.4 ±7.31	75.4 ±7.01	0.02*
75	78.43 ±6.96	75.2 ±4.48	73.4 ±7.27	0.010*
90	80 ±8.33	77.93 ±8.97	75.3 ±6.40	<0.001*
105	79.16 ±8.95	77.16 ±7.28	74.96 ±7.46	0.128
120	82.7 ±12.82	78.16 ±5.51	76.43 ±8.66	0.06

Table 4 presents the comparison of the partial pressure of oxygen measured during anesthesia between the groups. There was a statistically significant difference between the pO_2_ among the participants measured at 30 minutes of surgery and post-extubation. Similarly, when comparing between the groups, there was a statistically significant difference at 30 minutes of surgery and post-extubation between Group A, Group B, and Group C. The arterial oxygenation measured 30 minutes after intubation was better in PEEP 10 (154.9 ±29.88) and PEEP 5 (142.26 ±24.7) than in PEEP 0 (128.18 ±13.3) (p=0.002). Similarly, arterial oxygenation measured 30 minutes after extubation was also better in PEEP 10 (115.46 ±15.2) and PEEP 5 (105.1 ±8.28) than in PEEP 0 (97.07 ±9.90) (p<0.001).

**Table 4:** Comparison of partial pressure of oxygen during anesthesia between the groups (n=90) *Statistically significant (p<0.05 indicates statistical significance) SD: standard deviation

Timeframe	Partial pressure of oxygen, mmHg, mean ±SD			P-value	Group A vs. Group B	Group A vs. Group C	Group B vs. Group C
	Group A (n=30)	Group B (n=30)	Group C (n=30)				
Pre-induction	99.88 ±8.63	103.02 ±9.12	99.52 ±5.94	0.184	0.158	0.827	0.141
30 minutes	128.18 ±13.3	142.26 ±24.7	154.9 ±29.88	0.002*	0.016*	0.00*	0.042*
Post-extubation	97.07 ±9.90	105.1 ±8.28	115.46 ±15.2	<0.001*	0.010*	<0.01*	0.008*

**Figure 2:**
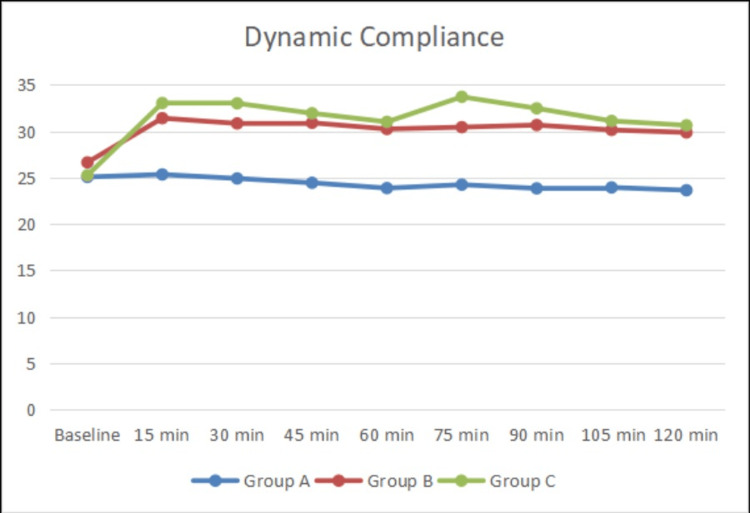
Line diagram of dynamic compliance variability between Group A, Group B, and Group C (n=90)

Table 5 shows the dynamic compliance between the three groups at various time intervals during anesthesia. Dynamic compliance was higher in Group C and Group B than in Group A. The increase in dynamic compliance was statistically significant between the groups. Figure 2 illustrates the significance between the three groups.

**Table 5:** Comparison of dynamic compliance during anesthesia between the groups (n=90) *Statistically significant (p<0.05 indicates statistical significance) SD: standard deviation

Time, minutes	Dynamic compliance, ml/cmH_2_O, mean ±SD			P-value
	Group A (n=30)	Group B (n=30)	Group C (n=30)	
Baseline	25.06 ±4.43	26.63 ±7.0	25.24 ±5.97	0.53
15	25.33 ±5.95	31.4 ±6.89	33.03 ±5.67	<0.001*
30	24.9 ±4.69	30.83 ±6.37	33 ±5.48	<0.001*
45	24.43 ±3.85	30.9 ±6.59	31.93 ±6.10	<0.001*
60	23.86 ±3.93	30.23 ±6.2	31 ±8.13	<0.001*
75	24.23 ±3.87	30.43 ±6.53	33.7 ±6.60	<0.001*
90	23.83 ±3.70	30.66 ±6.75	32.46 ±6.89	<0.001*
105	23.93 ±3.28	30.13 ±5.82	31.1 ±6.95	<0.001*
120	23.63 ±3.29	29.86 ±5.74	30.63 ±6.91	<0.001*

Table 6 details the amount of blood loss during surgery between the three groups. The blood loss was highest in Group A followed by Group B and the lowest in Group C. The intraoperative blood loss was lower in Group C (329.66 ±93.93) and Group B (421.16 ±104.52) than in Group A (466.66 ±153.76). The difference in blood loss was statistically significant between the groups.

**Table 6:** Comparison of blood loss during anesthesia between the groups (n=90) *Statistically significant (p<0.05 indicates statistical significance) SD: standard deviation

Blood loss, ml, mean ±SD						
Group A (n=30)	Group B (n=30)	Group C (n=30)	P-value	Group A vs. Group B	Group A vs. Group C	Group B vs. Group C
466.66 ±153.76	421.16 ±104.52	329.66 ±93.93	<0.001*	0.18	0.01*	<0.001*

## Discussion

This randomized controlled trial compared the effects of three different PEEP levels on respiratory parameters in patients undergoing spine surgery in the prone position under general anesthesia. The key findings indicate that higher PEEP levels (5 cmH_2_O and 10 cmH_2_O) are associated with improved arterial oxygenation, better dynamic compliance, reduced intraoperative blood loss, and stable hemodynamic parameters compared to no PEEP. PEEP demonstrated significant progress in several areas related to perioperative care in prone position procedures. First and foremost, its use significantly increased arterial oxygenation both during and after surgery, highlighting its critical function in maximizing respiratory function amid the difficult dynamics of prone placement. Maintaining alveolar recruitment and reducing ventilator-induced lung damage led to PEEP becoming an essential tool for pulmonary function maintenance, which in turn promoted improved oxygen exchange and alveolar stability [14,15].

Moreover, the judicious use of PEEP demonstrated a significant reduction in intraoperative blood loss, showcasing its multifaceted benefits beyond respiratory enhancement. This reduction in hemorrhagic complications may be attributed to its role in preserving hemodynamic stability, potentially mitigating factors predisposing to excessive surgical bleeding. The ability of PEEP to bolster dynamic compliance further underscores its utility in promoting respiratory mechanics, thereby facilitating efficient ventilation while mitigating undue stress on the pulmonary system [14,16]. Conversely, while higher levels of PEEP correlated with a notable decrease in MAP, this observation must be interpreted within the context of its broader physiological implications. Despite the reduction in MAP, the overarching benefits conferred by optimized respiratory function and reduced intraoperative blood loss underscore the net positive impact of PEEP utilization in prone-position surgeries [17-19].

The nuanced titration of PEEP levels emerges as a critical consideration, necessitating a delicate balance between alveolar recruitment and potential hemodynamic effects. Tailoring PEEP levels to individual patient factors, including body habitus and lung condition, remains imperative to optimize outcomes while minimizing adverse sequelae. Utilizing modalities such as respiratory compliance assessment holds promise in refining PEEP titration strategies, affording clinicians greater precision in achieving optimal respiratory dynamics [20,21]. Transitioning from the supine to the prone position, particularly with devices like the Wilson frame that restrict abdominal movement, poses challenges such as increased peak airway pressure (Ppeak) and decreased dynamic compliance (Cdyn). To address this, PCV was employed in our study to mitigate the rise in Ppeak associated with prone positioning on the Wilson frame. Despite ongoing debates regarding its impact on oxygenation, PCV has consistently demonstrated a reduction in Ppeak compared to volume-controlled ventilation (VCV) in patients with acute respiratory distress syndrome [21-24].

In cases of diminished lung compliance, elevated airway pressures are necessary for adequate ventilation; however, this may compromise venous return to the heart, leading to systemic venous pressure elevation, including within the epidural vein due to its connection to the inferior caval vein via a valveless venous system. Such hemodynamic alterations can predispose to heightened surgical bleeding during prone positioning, potentially culminating in neurological complications stemming from reduced perfusion pressure to the spinal cord [18,23,25]. The implementation of PEEP emerges as a pivotal strategy in preserving alveolar recruitment and safeguarding against ventilator-induced lung injury, particularly amidst PCV. By facilitating pressure equilibration during ventilation, PEEP aids in maintaining open alveoli, thereby mitigating the risk of atelectrauma and bolstering gas exchange efficiency [14,21,26].

Our study underscores the critical role of PCV and PEEP in optimizing respiratory mechanics during prone positioning, offering a means to mitigate the challenges associated with increased airway pressure and diminished lung compliance. Through meticulous ventilation strategies, we aim to not only enhance patient safety but also advance the perioperative management of prone-position surgeries, ultimately improving clinical outcomes and minimizing complications [21,24,27]. Elevated intrathoracic pressure during prone positioning poses a risk of inferior vena cava (IVC) obstruction, impairing venous return to the heart and potentially precipitating hemodynamic instability. This phenomenon may culminate in decreased stroke volume, although alterations in HR and MAP may be mitigated by compensatory increases in systemic and pulmonary vascular resistance. Notably, our observations revealed no significant disparity in MAP and HR despite the increase in intra-abdominal pressure (IAP) [25,28].

The rise in IAP concurrently elevates pressure within the IVC, transmitting these hemodynamic changes to the valveless epidural vessels. This cascade of events can manifest as visual impairment within the surgical field, underscoring the intricate interplay between intra-abdominal dynamics and surgical outcomes. The variability in blood loss, contingent upon the extent of surgery, underscores the multifactorial nature of intraoperative hemorrhage, with estimated losses approximating 10 ±30 mL/kg. Moreover, our study elucidated a balanced distribution of surgical procedures across study groups, reflecting a representative sample conducive to robust comparative analyses. Through meticulous elucidation of these hemodynamic nuances, we aim to enhance our understanding of the intricate physiological responses underlying prone-position surgeries, ultimately optimizing patient care and surgical outcomes [14]. Finding the right PEEP level is important because ventilation strategies need to fit each person's unique body type, whether they are lean or obese [26].

While the study provides valuable insights, it has certain limitations. The sample size was relatively small, and the study was conducted in a single center, which may limit the generalizability of the findings. Additionally, long-term outcomes related to PEEP application were not assessed. Future studies with larger sample sizes and multicenter designs are needed to confirm these findings and explore the long-term effects of PEEP on patient outcomes.

## Conclusions

This study demonstrates that higher levels of PEEP (5 and 10 cmH_2_O) during prone positioning in spine surgery significantly improve arterial oxygenation, dynamic compliance, and hemodynamic stability while reducing intraoperative blood loss. These findings emphasize the importance of optimizing ventilatory support to enhance patient outcomes during prone position surgeries. Further research is warranted to establish optimal PEEP levels and explore the long-term benefits of PEEP in various surgical contexts.
